# Malignancy in Membranous Nephropathy: Evaluation of Incidence

**DOI:** 10.1155/2017/8409829

**Published:** 2017-07-16

**Authors:** Basil Alnasrallah, John F. Collins, L. Jonathan Zwi

**Affiliations:** ^1^Department of Nephrology, Auckland City Hospital, 2 Park Drive, Grafton, Auckland 1023, New Zealand; ^2^Department of Pathology, Auckland City Hospital, 2 Park Drive, Grafton, Auckland 1023, New Zealand

## Abstract

**Background:**

Membranous nephropathy (MN) can be associated with malignancy. However, the relative risk for malignancy remains unclear. It has been reported that higher numbers of inflammatory cells seen in the glomeruli at biopsy correlate with the occurrence of malignancy in patients with MN and might be used to direct screening.

**Methods:**

We examined the occurrence of malignancy in 201 MN patients in Auckland, New Zealand. We also examined the pathology of renal biopsies from 17 MN patients with malignancies and compared the number of inflammatory cells per glomerulus with matched control patients with MN but no malignancy.

**Results:**

40 malignancies were identified in 37 patients, 28 of which occurred after the MN diagnosis. The standardized incidence ratio (SIR) was 2.1 (95% CI, 1.3–2.85) which was similar between patients ≥ 60 years and those <60 years. The median number of inflammatory cells per glomerulus did not differ between MN patients with and without malignancy at 1.86 (IQR, 1.17–2.7) and 2.07 (IQR, 1.17–3.65), respectively (*p *value 0.56).

**Conclusions:**

The relative risk of malignancy in MN patients was similar across different age groups. The number of inflammatory cells per glomerulus did not differentiate between MN patients with and without malignancies.

## 1. Introduction

Membranous nephropathy (MN) is a pathologic term that defines a specific glomerular lesion characterized by thickened glomerular capillary walls due to subepithelial deposition or in situ formation of immune-complex deposits [1]. The formation of these immune deposits leads to injury and impairment of glomerular basement membrane [2].

MN remains one of the most frequent glomerular diseases that cause nephrotic syndrome, accounting for around a third of renal biopsies in nondiabetic adults [3, 4]. About 75% of MN cases are idiopathic with no identifiable immunological stimulus and the remaining cases are secondary to a wide variety of primary causes, including neoplasia, infections, autoimmunity, and drugs [5, 6].

The link between MN and malignancy has been reported as early as 1966 by Lee et al. [7] and this association was confirmed later in a number of observational studies. However, the actual risk of malignancy has been shown to be quite variable between different studies, ranging from 2- to 10-fold increase relative to a matched population [8–11]. The malignancies most frequently associated with MN are solid tumors, including lung, gastrointestinal, and prostate carcinomas [9, 10, 12, 13].

This association has led to recommendations for regular screening for malignancy in MN patients around the time of diagnosis, especially when no obvious primary cause is identified [14]. The risk of malignancy in the following years is even less clear. In a population based analysis, the increased risk of malignancy associated with glomerulonephritis diminished after the first year of diagnosis and was not seen beyond 5 years [11].

A more recent study reported a significantly high annual risk of malignancy in patients with MN, which persisted up to 15 years after the diagnosis of MN [9]. This finding could impact on clinical practice and patients' outcomes, as more rigorous screening for malignancy during follow-up might be warranted. Identifying risk factors for malignancy can assist targeting at risk patients for future screening. However, only older age has been consistently found to be associated with malignancy risk [8–10]. Histological findings such as strong immunofluorescence staining with anti-immunoglobulin (Ig) G1 and G2 antibodies have been linked to malignancy-associated membranous nephropathy whereas IgG4 deposits were found predominantly in the idiopathic form of the disease [15, 16]. However, both of these findings have been challenged [17] and a recent review did not find a reliable immunoglobulin glomerular deposition pattern to differentiate between the two groups [18]. The high number of inflammatory cells infiltrating the glomeruli has been previously reported to be strongly associated with malignancy in MN patients compared to those without malignancy [10]. To our knowledge this has not been further validated or challenged. However, if true it could be a useful additional guide for clinicians to target their screening without the need for further staining.

We undertook a retrospective study of 201 MN patients to try to clarify the incidence of malignancies in patients with MN in Auckland, New Zealand, at the time of diagnosis and during follow-up and to examine the associated factors.

## 2. Materials and Methods

Ethical approval was obtained through the Northern B Health and Disability Ethics Committee of the Ministry of Health in New Zealand. Ethics approval number is* 15/NTB/196*.

### 2.1. Identification of Patients

Patients were identified from the renal biopsy records of the Department of Pathology in Auckland City Hospital. All adult patients (≥18 years old) in whom the diagnosis of MN was made on renal biopsy between January 2003 and December 2013 were included. Baseline data at the time of renal biopsy were collected which included the following: age, sex, eGFR (4-variable Modification of Diet in Renal Disease Study equation), serum albumin level by BCG dye-binding method, degree of haematuria (as per urine RBC*∗*10^6^/L, no haematuria if <10, microscopic haematuria if between 10 and 100, and gross haematuria if >100), and proteinuria (g/day) or protein/creatinine ratio (mgs/mmol).

### 2.2. Definition of Observation Period

The observation period of patients was defined as the time between kidney biopsy and December 31, 2015; death; or loss to follow-up.

### 2.3. Data for Kidney Biopsies

The pathology slides of renal biopsies in MN patients with malignancy diagnosed around the time of MN (from 1 year before to 5 years after renal biopsy) were reviewed, excluding patients with nonmalignant primary causes of MN. A group of idiopathic MN patients without malignancy were matched in age and gender at a rate of 2 : 1 to the cases and reviewed. All the renal biopsies were examined by a renal pathologist (LJZ) who was blinded to the clinical and laboratory data of the patients. The diagnosis of MN was confirmed histologically and the number of inflammatory cells per glomerulus was reported.

### 2.4. Identification of Malignancy in Patients with MN

We linked the patients' records using the New Zealand National Health Index number with the New Zealand Cancer Registry (NZCR), which is a register established in 1948 of all primary malignant diseases diagnosed in New Zealand, excluding squamous and basal cell skin cancers. In addition, the clinical records of all patients were systematically reviewed by a Renal Fellow (BA) to confirm the presence or absence of malignancy. All malignancies diagnosed from 1998 (5 years before the first renal biopsy) to December 2015 (end of observation period) were recorded.

At the time of conducting the study, NZCR had full data of malignancies to the end of 2013. For the years 2014 and 2015, malignancies were recorded based only on systematic review of the clinical records.

32 malignancies were reported on NZCR between 1998 and 2013 in 29 patients and confirmed on clinical records (6 malignancies in 3 patients, 2 each). In addition, on review of clinical records for the same period, 2 malignancies in 2 patients were identified and had not been reported by NZCR.

For the years 2014 and 2015, 6 more malignancies in 6 patients were identified on clinical records and therefore we report on 40 malignancies in 37 patients.

### 2.5. Statistical Analyses

Standardized incidence ratios (SIRs) were calculated as ratios between observed and expected numbers for site-specific malignancies and all malignancies. For the SIR for all malignancies and their subanalyses, the individual annual expected rate for a matched population in gender and in 5-year age groups was added for every year of follow-up using the rate of that particular year as a reference.

For example, for a 64-year-old male who is followed up for 10 years from 2004, the expected national malignancy rate of the year 2004 for males aged 60 to 65 years was used for the first year, for the second year, the national rate of the year 2005 of the same group was used, and, for the third year, the 2006 rate for males aged 65 to 70 years was used and so on. This was to provide the best representation of the expected malignancy risk and to minimize underestimation of the expected risk of malignancy, as malignancy risk increases with age.

Independent-samples *t*-test was used with continuous variables and chi-square test was used with categorical variables. Median and interquartile ranges (IQR) are reported by the weighted average method.

## 3. Results

The clinical records of 201 MN patients were reviewed, MN was classified by the treating nephrologist to be idiopathic in 158 patients (78.6%), and this was made after excluding primary causes of MN. Investigations for malignancy, immunological disorders, and hepatitis B and C were universal in this cohort. However, the details of some of the tests were not available to us for many of the patients. Testing for phospholipase A2 receptor (PLA2R) antibodies was not available in New Zealand at the period of patients' diagnoses (2003–2013) and therefore was not done in any of the patients. The diagnosis of secondary MN was made in 43 patients (21.4%). MN was classified as related to malignancy in 4 patients (2%) and to nonmalignant primary causes in 39 patients (19.4%) (30 patients, systemic lupus erythematosus; 5 patients, hepatitis B; 2 patients, graft-versus-host disease, 1 patient; visceral leishmaniasis, 1 patient; connective tissue disease-NOS). It should be emphasized that there is no clear definition for malignancy related MN in the literature; few criteria have been proposed to confirm a truly causal relationship between the two [12, 13].

The overall follow-up period from the date of biopsy was 1384 patient-years. Baseline characteristics at the time of renal biopsy are shown in Table 1.

### 3.1. Malignancies

40 malignancies were identified in 37 patients (6 malignancies in 3 patients, 2 each) with a prevalence of 18.4%. Of the patients with nonmalignant primary causes of MN, only one had a malignancy during the observation period.

12 out of the 40 malignancies (30%) had been diagnosed before the diagnosis of MN in 10 patients (2 malignancies in each of 2 patients). The median time from diagnosis of these malignancies to renal biopsy was 65 months (IQR, 8.75–76.25). 28 malignancies were diagnosed after MN diagnosis in 27 patients, 4 of these were in malignancy related MN and were diagnosed within 6 months after renal biopsies. The median time from the diagnosis of MN to the diagnosis of malignancy was 40 months (IQR, 14–67.5). When the 4 cases of malignancy related MN were excluded, the median time to the diagnosis of malignancy was 44 months (IQR, 29–73.25). A de novo malignancy after MN was not diagnosed in any of the patients with malignancy prior to MN.

### 3.2. Risk Factors

Only age was a significant risk factor for malignancy as in Table 2.

### 3.3. *Immunosuppressive* Therapy

The use of* immunosuppressive* therapy (cyclophosphamide and/or calcineurin inhibitors and/or mycophenolate and/or azathioprine) for more than 3 months was not different between MN patients who did and did not develop malignancy at 42.9% (9 patients) and 42.7% (50 patients), respectively. However, when only looking at the use of cyclophosphamide, more patients had 3 months or more of therapy in the malignancy group at 20.5% (8 patients) compared to 13.8% (13 patients), respectively,* p *value < 0.001. The mean cumulative dose to cyclophosphamide was 19.8 grams (SD +/− 12.2), with no significant difference between the 2 groups.

### 3.4. During Follow-Up

After excluding patients with malignancy prior to MN diagnosis, the follow-up period was 1284 person years from the date of renal biopsy. The Standardized Incidence Ratio (SIR) for cancer was significantly raised at* 2.1 (95% CI, 1.3–2.85)*, it was* 1.925 (95% CI, 1.06*–*2.79)* in males and* 2.51 (95% CI, 0.87*–*4.15)* in females. When the cases of malignancy related MN were excluded, the SIR remained significantly elevated at* 1.78 (95% CI, 1.06*–*2.49)* in the follow-up period.

**Figure 1:**
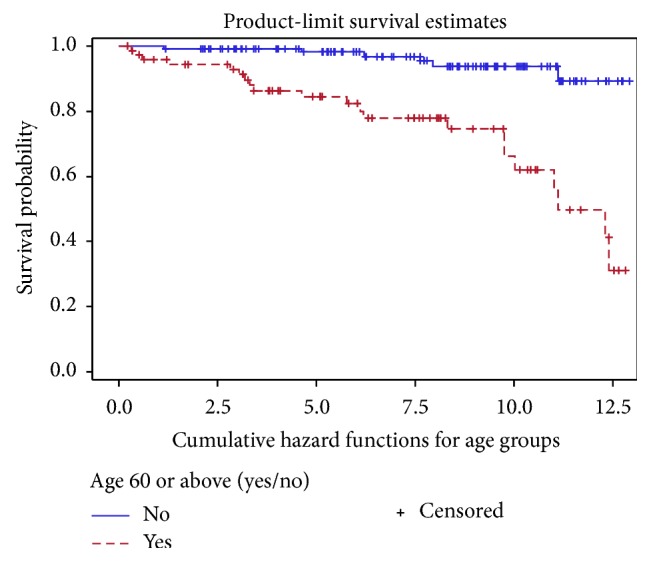
Malignancy-free survival in patients ≥ 60 years of age and those <60 years.

Interestingly, the SIRs for patients who are ≥60 years and those <60 years were similar at* 2.08 (95% CI, 1.16*–*2.99)* and* 2.06 (95% CI, 0.63*–*3.49)*, respectively. This is despite a higher risk of malignancies in the older group and subsequently a significantly lower malignancy-free survival rate (*p* < 0.001), as seen in Figure 1.

When broken down by time from biopsy to 0–5 years and >5 years, the SIR was significantly high for the first 5 years at* 2.3 (95% CI, 1.29*–*3.4)* and lost statistical significance after that at 1.7 (95% CI, 0.58–2.77).

### 3.5. Symptoms

For the 27 patients who were found to have malignancy after the MN diagnosis, the symptomatic status prior to the diagnostic test for malignancy was known in 26 of the 28 malignancies (92.9%). 24 of 26 malignancies (92.3%) were symptomatic prior to investigations. The list of symptoms is shown in Table 3.

### 3.6. SIR for Types of Malignancies

The SIR for lung malignancy was significantly increased at* 8.9 (95% CI, 2.73*–*15)*. All of these happened in patients with a history of heavy smoking. The median time from MN diagnosis to the diagnosis of lung malignancy was 35 months (IQR. 14.5–61.5). The SIRs for colorectal and prostate malignancies in males were also high but not statistically significant at 4 (95% CI, 0.08–7.92) and 2.51 (95% CI, 0.5–4.5), respectively.

### 3.7. Review of Renal Biopsy Tissue

There were 20 patients with malignancy diagnosed between 1 year before and 5 years after MN diagnosis on renal biopsy. Two of these patients had the malignancy diagnosed prior to the biopsy and 18 had the malignancy diagnosed after. We attempted to obtain the pathology slides of these patients and compare them with matched controls with no malignancy at a ratio of 1 : 2.

**Figure 2:**
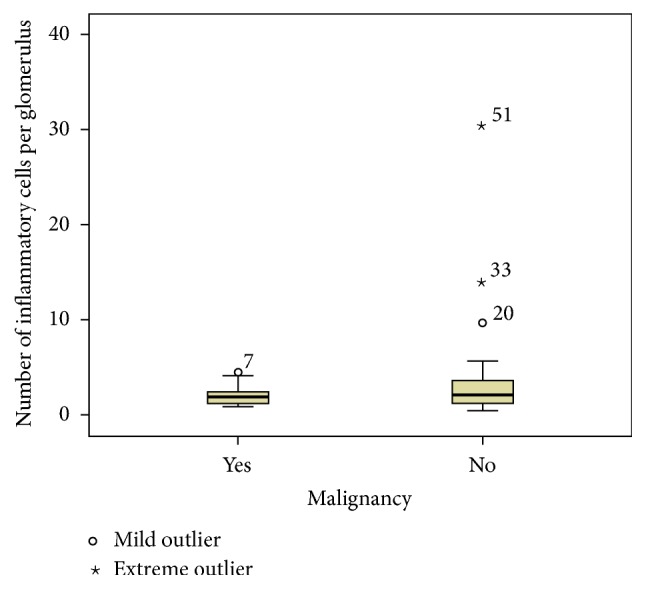
The median number of inflammatory cells per glomerulus in patients with and without malignancies.

17 biopsies from patients with malignancies were reviewed (3 could not be retrieved). All of these patients had 10 or more glomeruli on the biopsy with a median interval between the biopsy and the malignancy of 18 months (IQR, 4.5–41). 37 biopsies from patients with no malignancies were reviewed (3 could not be retrieved). The median numbers of glomeruli per biopsy in patients with and without malignancies were similar at 15 (IQR, 13–25.5) and 15 (IQR, 9–23), respectively. The median number of inflammatory cells per glomerulus was not different between the 2 groups at 1.86 (IQR, 1.17–2.7) in patients with malignancy and 2.07 (IQR, 1.17–3.65) in those without, *p* value 0.56, as in Figure 2.

The number of glomeruli analyzed on different levels was 917 (311 in the malignancy group and 606 in the control group).

A univariate analysis on Pearson test showed no correlation between the number of inflammatory cells per glomerulus and the period between cancer diagnosis and renal biopsy,* p *value 0.44. The median number of inflammatory cells per glomerulus was not different in the 6 patients with malignancies within 12 months of renal biopsy compared to the remaining 11 patients with malignancies at 1.01 (IQR, 0.87–1.95) and 2 (IQR 1.8–3), respectively,* p *value 0.3. No correlation between the total number of inflammatory cells in the biopsy or the inflammatory cells per glomerulus has been identified with any of the clinical variables (age, proteinuria, haematuria, eGFR, or serum albumin).

## 4. Discussion

This study provides strong evidence for a twofold increased risk of malignancy in MN patients in the follow-up years when compared to a matched population. The SIR of 2.1 is similar to that reported by Bjørneklett et al. at 2.25 who had a median follow-up of 6.2 years [9]. The minor disparity might be related to the different methods used. In the present study, the expected risk was calculated taking into account the annual change of risk, which minimized overestimation of the SIR.

MN patients are regularly screened for malignancy around the time of their diagnosis, and given the known association there might be a lower threshold for future screening compared to general population, which raises the possibility of detection bias. Lefaucheur et al. attempted to address this by identifying those patients with symptomatic malignancies and found that 11 patients (52%) were symptomatic, and the SIRs were raised at 7.1 (95% CI: 3.4–13) in males and 4.4 (95% CI: 0.5–16) in females [10]. However, this was a subsidiary analysis of a group with a very high risk at the start (10-fold). During the follow-up after MN diagnosis in our study of 88 months, the number of symptomatic malignancies before diagnostic testing was high at 24 (92.3%). This might relate to the long follow-up as MN patients usually have screening for malignancy around the time of MN diagnosis, especially if no primary cause is found [1], but not routinely investigated during follow-up unless indicated or symptomatic. Therefore, the detected malignancies in our study are likely to be representative of the actual risk in these patients and not of better surveillance.

The data on long-term risk of malignancy in MN patients remains limited. In the present study, the risk of new malignancies decreased and lost significance beyond 5 years after the diagnosis of MN. After excluding patients with previous malignancies we had 1284 patient-years, which makes this study the largest of its kind.

Older age (≥60 years) has again been shown to be a strong risk factor for malignancy in MN patients; however, the SIRs were almost identical between old and younger patients. This should be emphasized to patients and clinicians when describing the malignancy risk in MN patients.

In the patients receiving cyclophosphamide for more than 3 months in our study, there was a higher rate of malignancy in the follow-up years. van den Brand et al. reported a significant increase in malignancy risk in MN patients receiving cyclophosphamide from 0.3% to 1% [19]. Cyclophosphamide has been linked historically to bladder malignancy [20] and haematological malignancies [21] especially when the mean cumulative dose is >36 g. However, none of the 44 MN patients who had cyclophosphamide in this study developed either. This might be partly explained by the fact that the mean cumulative dose in our cohort was not particularly high at 19.6 g, which falls within the current recommendations for managing idiopathic MN [14].

Lung malignancy has been reported to be the commonest malignancy in MN patients at 26% [8]. In our study the SIR was high at 8.9 and this was within the range of previously reported rates, 3.33 (95% CI: 0.91–8.52) [3] and 31.9 (95% CI: 12.8–65.7) [10]. All lung malignancies in our cohort were in heavy smokers. We thus support the recommendation that a Computed Tomography of the chest be undertaken for smokers with MN as proposed by Pani et al. [22]. However its value has not been prospectively examined.

Efforts have been made to identify histological markers in renal biopsies of MN patients which predict malignancy. Earlier papers suggested that different IgG subclass pattern of deposition could be helpful. Ohtani et al. reported a significantly more intense IgG1 and IgG2 deposition in malignancy-associated MN compared with idiopathic MN [15]. Later on, Qu et al. found no significant difference in IgG1 deposition and a small but significantly more intense IgG2 deposition in malignancy related MN compared to idiopathic MN [16]. Qu et al. also found that the IgG4 deposition was significantly different between the 2 groups, being absent in 88% of malignancy related MN compared to 14% of idiopathic MN. More recently, Lönnbro-Widgren et al. showed no difference between idiopathic MN and malignancy related MN in IgG1 or IgG2 deposition and reported a difference only in the IgG4 deposition between the two at 65% and 31%, respectively [17]. Murtas and Ghiggeri reported on a recent review that there was no reliable deposition pattern to differentiate between the two entities [18].

In their paper, Lefaucheur et al. reported that MN patients with malignancies had a significantly higher number of inflammatory cells in the glomeruli compared with matched idiopathic MN patients. The number of inflammatory cells was on average more than double that of the matched controls, and 8 inflammatory cells was the best cut-off value to distinguish between the 2 groups, with a specificity of 75% and a sensitivity of 92% (*p* = 0.001). This has not been further examined.

In the present study, there was no relation of the number of inflammatory cells in the glomeruli to the malignancy status or to the interval between the renal biopsy and the malignancy diagnosis. We had 3 patients in our cohort with 8 inflammatory cells or more per glomerulus on their renal biopsies and none of these patients had malignancy.

The exact mechanism underlying the association between MN and malignancy remains a matter of debate. Nevertheless, few mechanisms have been proposed [23, 24]. Antibodies may be generated against an antigen on the malignant tissues similar to an endogenous podocyte antigen, thereby leading to in situ glomerular immune-complex formation. Alternatively, shed tumor antigens may form circulating immune complexes that become trapped in the capillary wall. These complexes may initially form in a subendothelial location where the immune clearance is limited [25], dissociate, and reform in a subepithelial position. Another possibility is an extrinsic process such as an oncogenic virus or an altered immune function that causes both malignancy and MN.

A major argument against a causal association between malignancy and MN stems from the fact that they are both more common in older individuals which might merely represent two coincidental disease processes [24]. However, in our study we demonstrated clearly that this association persists across the age groups.

## 5. Conclusion

The relative risk for malignancy after the diagnosis of MN is more than twice that of a matched population and is similar across age groups. This increased risk is only found in the first 5 years of follow-up. The increased risk does not appear to relate to better surveillance, as it remains significantly high even for patients only with malignancy-specific symptoms prior to investigations. The risk of lung malignancy is particularly high at almost 9-fold. The number of inflammatory cells per glomerulus was not a helpful tool to differentiate between MN patients with and without malignancy.

## Figures and Tables

**Table 1:** Cohort characteristics at time of MN diagnosis.

Age (years)	56 (41–65)
Male	117 (58.2%)
Follow-up (months)	88 (45–117)
Serum albumin g/L	29 (23–35)
Proteinuria g/d	5 (2.6–9.55)
eGFR ml/min	70 (43.2–97.5)
Haematuria	84, no haematuria (43.3%)
	51, microscopic haematuria (26.3%)
	59, gross haematuria (30.4%)

Results are expressed as median (interquartile range) or as percentage.

**Table 2:** Clinical and laboratory data at time of kidney biopsy in patients with and without malignancy.

	Without malignancy	With malignancy	*p* value
Age (years)	53 (38.5–63.5)	65.5 (58.5–72)	<0.001
Proteinuria (g/d)	5 (2.7–8.85)	6.7 (2.05–10)	NS
Albumin (g/L)	30 (23–36)	26.5 (21.25–35)	NS
eGFR (ml/min)	70 (43–97)	65 (43.4–99.5)	NS
Haematuria			
(i) Nil	68 (43%)	16 (44.4%)	NS
(ii) Microscopic	41 (26%)	10 (27.8%)	
(iii) Gross	49 (31%)	10 (27.8%)	

Results are expressed as median (interquartile range) or as percentage; NS: *p* value > 0.05.

**Table 3:** Types of malignancy and symptoms in new malignancies after MN.

localization of tumour	Histology	Age	Sex	Months after renal biopsy	Symptoms before malignancy diagnosis (Y: yes/N: no)
Rectal C20	Adenocarcinoma	66	m	1	Y, rectal bleeding
Pancreatic C259	Adenocarcinoma	65	f	1	Y, abdominal pain
Lung NSCLC C349	Not done	75	f	4	Y, dyspnea
Lung NSCLC C343	SCC	57	f	6	N
Cervical D069	CIN III	34	f	7	Y, vaginal bleeding
Colon C187	Adenocarcinoma	82	m	12	Y, symptomatic anaemia
Lung NSCLC C341	SCC	68	m	14	Y, haemoptysis
Prostate C61	Adenocarcinoma	62	m	17	Unknown
Left upper back C435	Metastatic melanoma	72	m	18	Y, skin lump
Lung NSCLC C341	SCC	74	m	27	Y, dry cough and dyspnea
Lung NSCLC C340	Adenocarcinoma	80	m	36	Y, chronic cough
Colon C19	Adenocarcinoma	74	m	37	Y, obstructive bowel symptoms
Lung NSCLC C343	Not done	74	m	37	Y, weight loss
Intestine C179	Neuroendocrine	72	f	41	Y, breast nodule
Unknown	Metastatic melanoma	79	f	41	Y, Dyspnea
Prostate C61	Adenocarcinoma	64	m	45	Y, Urinary voiding symptoms
Prostate C61	Adenocarcinoma	58	m	46	unknown
Intestinal C494	Liposarcoma	54	m	47	Y, Bloody diarrhoea
Unknown primary C269	metastatic adenocarcinoma	51	m	48	Y, Lower groin pain
Prostate C61	Adenocarcinoma	51	m	61	Y, Urinary voiding symptoms
Prostate C61	Adenocarcinoma	68	m	66	Y, Urinary voiding symptoms
Bile duct C240	Adenocarcinoma	64	f	69	Y, Jaundice
CLL C9110	CLL	55	f	76	Y, Cervical lymphadenopathy
Lung NSCLC C341	Adenocarcinoma	64	m	76	Y, Chest wall pain
Colon C182	Adenocarcinoma	61	m	101	Y, Change of bowel habit
Kidney C64	Clear cell carcinoma	55	f	108	N
Lung NSCLC C349	Adenocarcinoma	60	m	117	Y, Dry cough and dyspnea
Prostate C61	Adenocarcinoma	61	m	143	Y, Macroscopic haematuria
